# Customer Segmentation through Path Reconstruction

**DOI:** 10.3390/s21062007

**Published:** 2021-03-12

**Authors:** Santiago García Carbajal

**Affiliations:** Computer Science Department, Campus de Viesques, University of Oviedo, Office 1.b.15, Gijón, 33003 Oviedo, Asturias, Spain; sgarcia@uniovi.es; Tel.: +34-985-18-24-87

**Keywords:** path analysis, in-store behavior, customer clustering, indoor positioning, trajectory analysis, multilateration

## Abstract

This paper deals with the automatic classification of customers on the basis of their movements around a sports shop center. We start by collecting coordinates from customers while they visit the store. Consequently, any costumer’s path through the shop is formed by a list of coordinates, obtained with a frequency of one measurement per minute. A guess about the trajectory is constructed, and a number of parameters are calculated before performing a Clustering Process. As a result, we can identify several types of customers, and the dynamics of their behavior inside the shop. We can also monitor the state of the shop, identify different situations that appear during limited periods of time, and predict peaks in customer traffic.

## 1. Introduction

Understanding shoppers behavior is a hot topic in the management field. Recently, the first large-scale trajectory data set for shopper behavior understanding has been collected [1], reporting results for more than ten million shoppers in Germany. Some empirical patterns about in-store behaviors have been described and confirmed in the past [2], while others like the correlation between longer in-store travel distance and unplanned spending are still controversial [3]. In any case, the importance of the store’s layout and its influence on the customer’ s experience, and the utility of these data in order to obtain a valuable segmentation of the customers is massively accepted [4,5,6]. Our work is focused on the study and segmentation of the different types of customer behavior that we can identify based on the data from their movements along the store, without using any information about their purchases.

Cluster Analysis [7] is one of the vast group of Data Mining Techniques [8]. In Business Intelligence, the goal is to segment a customer database so that customers within clusters are similar, and as different as possible to customers in other clusters. Intra- and Inter-Cluster Similarity is measured in terms of some kind of clustering distance, for example, the sum of squared error (SSE) [9]. Clustering allows deep interpretation with many implications about which customers should be targeted with the particular offers most likely to attract them back to the store and to spend more money on their subsequent visits.

When talking about automatic classification of customers in general, we have found a similar approach applied to the field of Electrical Tariff Offer. Some examples are [10,11,12]. Most of these works use data related to user’s electricity consumption to determine different types of customers, and to obtain some kind of User Load Profiling. In [13], K-means algorithm is used to cluster customers based on their sales data records.

Unlike others, our approach is not based on information about the shopping behaviors of users, but on partial data related to their movements inside the premises. Some previous works like that in [14] use Artificial Vision techniques to track multiple persons in an indoor environment, but we decided to use the huge amount of information available thanks to the massive use of portable devices like tablets or smartphones instead.

Below, we list the most similar works we have found in the literature of the field.

The work in [15] is probably the first reference to a customer positioning Bluetooth-based system. The authors prove Bluetooth to be a good low-cost alternative for indoor environments. Some spatio-temporal analysis of the behavior of individuals is performed in the study. Nevertheless, the initial setting of the system, as it is described, is far from being easy to automate, and the reported detection ratio was around 10%, which was not acceptable for our needs. In our case, having WiFi enabled on the smartphone is enough to be detected and tracked by our system. In this sense, we could find no published study describing the percentage of people who keep the WiFi of their mobile device on, but in view of the number of trajectories that our system captures in short time intervals, we can say that it is clearly higher than that value. This study is based on data from trajectories captured along one month of commercial activity.

In [16], k-medoids algorithm [17] is used to study a huge dataset of paths recorded inside a supermarket. This is the most similar study we have found in the field literature. The main differences between the work of Larson, Bradlow, and Fader and this work are listed below.

- The clustering algorithm: we do not let the clustering process deal with spatial constraints. We take care of the process of conversion between detection points and valid trajectories, which are generated based on the lists of detection points, before performing any other calculation.
- Our method does not divide data into different subsets according to the time length of the paths. The user decides the maximum number of clusters they are interested in, and customers are then segmented according to all the numeric data that we can collect from their trajectories, with the length of the path being one of the variables used by the clustering algorithm.

More recently, Sorensen et al. [2] identify and validate customer patterns studying 654,000 transactions in 40 supermarkets, hypermarkets, and convenience and specialty stores. Data are analyzed using three behavioral metrics: store coverage, number of items bought, and trip duration. In our approach, no data about purchases is available, as this information must be private, and the study is performed only on the basis of the customer’s physical movements.

In [18], the authors study physical trajectories through the store and generate associated heat maps as we do, but customers are tracked using video cameras, and no customer segmentation is performed.

Customer trajectories have also been studied starting from an initial clustering made based on the gender (male, female). We could not perform such an initial clustering of our data as in the case we are studying, the fraction of customers using the toilets is known to be insignificant, and this is the information used to discriminate users in [19].

Moreover, in [20] the same authors perform automated clustering of customer paths based on two variables: case duration and the number of visited locations. We use a higher number of variables and let clustering process uncover relationships among them. We chose to use so many variables because they reflect all the information available about the customers, and some variable that is not useful at the moment from a segmentation point of view can be interesting for a different set of trajectories. This is the main strength of our approach: we are not trying to proof any previously described empirical pattern in the behavior of our customers. The goal is to collect as many data as possible about their trajectories, and detect the different types of behaviors lying undercover.

In [21], the authors study the connection between customer’s behavior and some of their psychographic characteristics like gender, age, or marital status.

Process Mining is used in [19,20,22] as a way to extract the topological structure of customer’s paths. We use heat maps instead to unveil the dynamics of people’s behavior inside the store. Additionally, in [22], customer behavior is analyzed after altering the position of some elements in the shop, which is something that is out of our goals, at least at this time.

This paper proposes a methodology for automatically classifying customers without the need to access actual purchase data, using only data relating to their behavior within the shop. To achieve this objective, we have defined a set of variables that we can calculate from the data coming from an indoor positioning system, and we have carried out an automatic clusterization procedure. Our results prove that it is possible to extract relevant knowledge from the customers’ positioning data.

The rest of the paper is organized as follows. In Section 2, we define the experimental setup for our work, the physical space we are dealing with, the data acquisition process, and the calculus we perform on the data. Section 3 describes generic considerations about Automatic Clustering Processes. We also discuss the application of such methods to our case study. In Section 5, we discuss our results obtained by applying the method to a month of commercial activity. The conclusions of this study are presented in Section 6, where future research lines are also analyzed.

## 2. Our Case Study

### 2.1. Physical Environment

**Figure 1:**
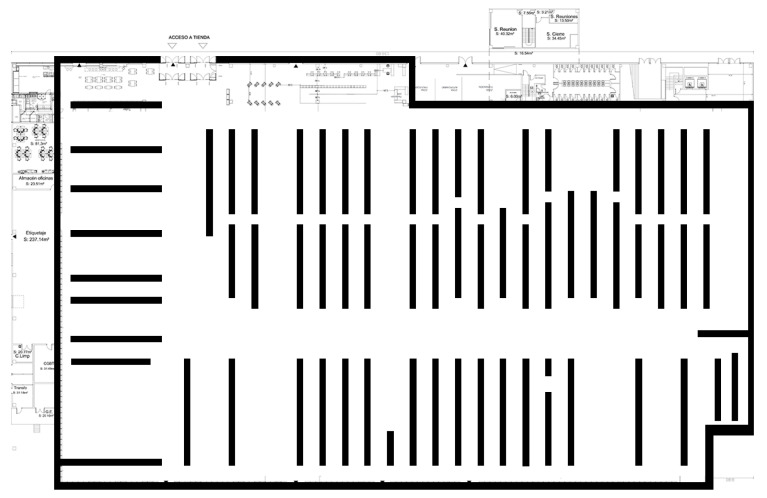
The shop: 138 × 90 m space with 30 different logistic sections.

The shop is a 138 × 90 m Sports Shop Center, divided into 30 different Logistic Sections, named “HEALTH”, “JCYCLING”, “CITYCYCLING”, “YOGA”, etc., depending on the kind of sport they are dedicated to. We consider corridors, fitting rooms, and some other small spaces which we generically call "UNDEFINED_AREA". Undefined areas are not taken into account when studying trajectories, see Figure 1.

We have also information about the positions where exhibitors and corridors are located. These data are important for trajectory reconstruction, because we only know where the users were at certain moments, and not every kind of movement is allowed inside the store. We use a well-known algorithm [23] to construct paths between the points where a user is sequentially detected, avoiding obstacles such as exhibitors, cashier machines, etc.

### 2.2. WiFi Positioning

**Figure 2:**
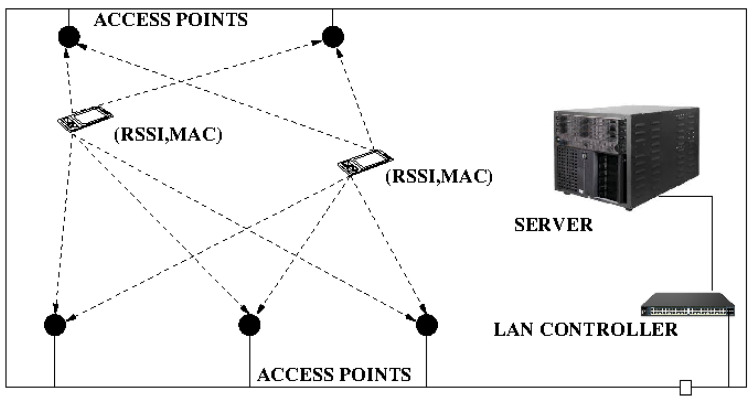
WiFi positioning system. First, the list of Access Points is determined. Second, for each Access Point, all the visible mobile devices are listed. The customer is then located using multilateration.

Inside buildings, WiFi is a good alternative to GPS, which is not available indoors. As WiFi access points already exist in many buildings, developing a WPS is quite straightforward. No additional installation is needed, as existing access points, exhibitors, and cash register systems can be used. The user does not necessarily have to connect to the local network, enabling WiFi on the smartphone is enough. The shop is equipped with multiple Access Points, or WiFi antennas, in order to ensure signal strength throughout the whole building. All of them are connected to a single wireless controller, which manages them independently. RSSI and the MAC address are the significant numeric values that we use to pinpoint any person inside the premises, see Figure 2.

The first step is to ask the Wireless LAN Controller (WLC) via SNMP protocol and find out the list of available Access Points. For each one of these Access Points on the list, another list containing all the devices that the Access Point can see (MAC Addresses) is built, see Figure 2.

RSSI data are given in decibels, so the second step is to convert these values into meters. To do that we need the signal strength and the frequency of the signal. The formula is a transformed form of FSPL.

(1) $$P\left(X\right)=10nlog\frac{d}{d_{0}}+20log\frac{4\pi d_{0}}{\lambda }$$

where

- P(X) is Path Loss at distance d
- *n* is the signal decay exponent
- *d* is the distance between transmitter and receiver
- $d_{0}$ is the reference distance. For us this value is 1 m
- λ is the wavelength of signal (2 Ghz = 0.125 m)
- $X_{\lambda }$ is the Fade Margin. It is system specific and has to be empirically calculated for each site. For buildings such as the the one we are testing our system in a common value of $X_{\lambda }$ is 10 dBm.

Multilateration is then used to pinpoint a user’s location in the shop with the available information. The multilateration problem can be written as follows:

(2) $$\left\{\begin{array}{cc} {\left(x_{0}-x\right)}^{2}+{\left(y_{0}-y\right)}^{2} & =r_{0}^{2}\\ {\left(x_{1}-x\right)}^{2}+{\left(y_{1}-y\right)}^{2} & =r_{1}^{2}\\ . & \\ . & \\ {\left(x_{n-1}-x\right)}^{2}+{\left(y_{n-1}-y\right)}^{2} & =r_{n-1}^{2} \end{array}\right.$$

where *n* is the number of Access Points (APs from now on) that can detect one device, D=(x,y) is the position of the device, $AccessPoint_{i}=\left(xi,yi\right)$ are the positions of the APs, and $r_{i}$ is the distance measured from the $i_{th}$ AP to device. This is a linearizable system. We just need to subtract the $i_{th}$ equation from all other n−1.

**Figure 3:**
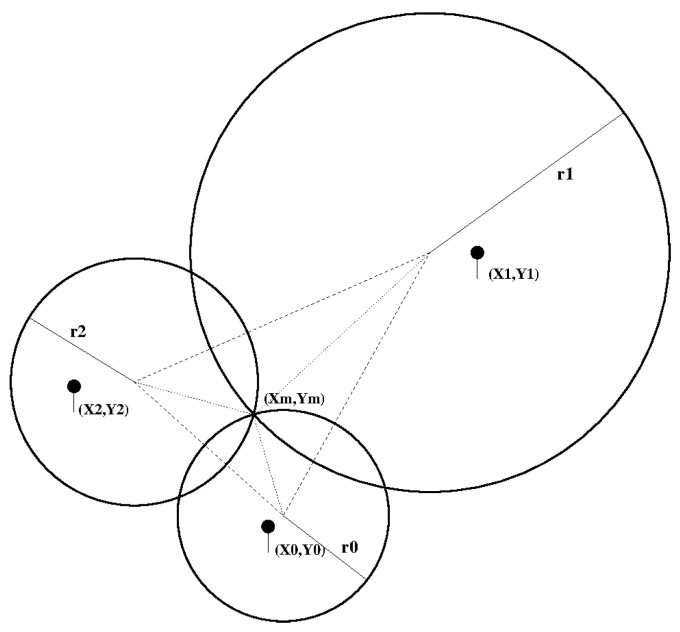
Ideal solution to a tri-lateration problem.

Ideally, all the circles would intersect at a single point, but in the real case measures are affected by error and they intersect at more than one point, see Figure 3. This list of points defines an area and the precision of the solution is given by the residual:

(3) $$res=\frac{\sum_{0}^{n-1}\sqrt{{\left(x_{i}-x_{m}\right)}^{2}+{\left(y_{i}-y_{m}\right)}^{2}}-r_{i}}{n}$$

$D_{m}=\left(x_{m},y_{m}\right)$ is the resulting estimated device position.

The accuracy of these methods depends on multiple factors, like wall reflections, or the number of available networks. At present, our positioning accuracy is under two meters, and we could even determine the current floor level, if it was the case. In the future, we are planning to apply the system to a multi-level mall, but for our current case study one floor is enough.

### 2.3. The Data

Our client has provided us with relevant data describing when and where each cell phone was detected inside the shop. Data corresponding to customer behavior comes in this way:

MAC_ADDRESS_0,2020-04-06 08:46:04 UTC,26.0,34.0,1
MAC_ADDRESS_0,2020-04-06 08:48:00 UTC,30.0,36.0,1
MAC_ADDRESS_0,2020-04-06 08:49:03 UTC,40.0,34.0,1
MAC_ADDRESS_0,2020-04-06 08:51:16 UTC,32.0,20.0,1
MAC_ADDRESS_0,2020-04-06 08:53:18 UTC,33.0,34.0,1
MAC_ADDRESS_0,2020-04-06 08:59:19 UTC,41.0,32.0,1
MAC_ADDRESS_0,2020-04-06 09:03:22 UTC,38.0,38.0,1

This is a huge file containing a MAC address, Coordinated Universal Time (UTC), (x,y) local coordinates inside the shop calculated with multilateration, and a sequence number that indicates if that MAC address has previously been detected inside the shop. Furthermore, a counter records on how many occasions, if this was the case. In the example, we can see a customer detected seven times. The customer has visited the shop once in the past. Actual MAC address has been hidden for confidentiality reasons.

We could try a direct translation of this list of coordinates into a full path, just by joining each pair of consecutive points with a straight line. However, shops are full of exhibitors and divided into corridors and areas, so such a direct transformation would result in an unrealistic path around the room. Consequently, we need to estimate the real trajectory, based on the data contained in this file, and taking into account the positions of obstacles. To that end, we chose an algorithm used in Maze Routing and Very Large-Scale Integrated circuit (VLSI) Design: Lee’s Algorithm [23].

**Figure 4:**
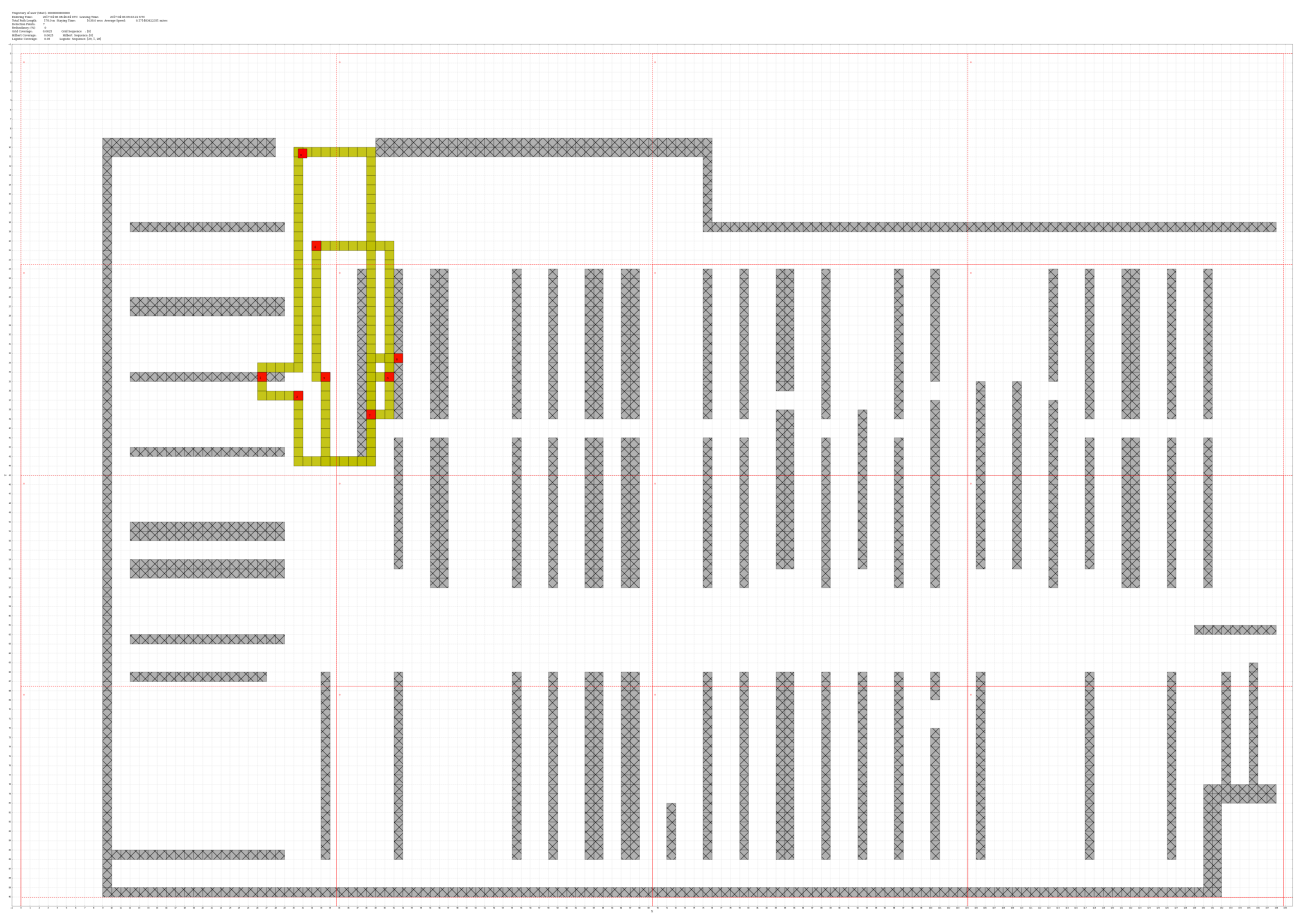
A short trajectory.

**Figure 5:**
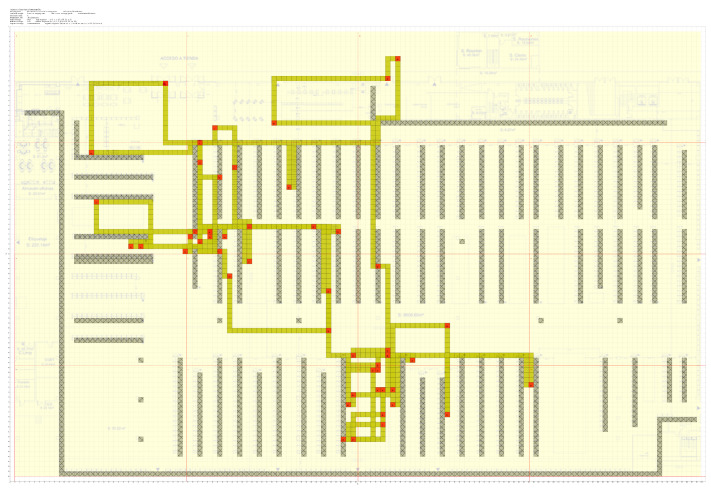
Long reconstructed trajectory.

In Figure 4 and Figure 5, the assumed behavior of a customer is shown for the short trajectory described above, and for a longer, realistic path along the store. Red squares represent points where the cell phone was detected. Yellow squares are generated by the algorithm, avoiding obstacles, which are represented as gray squares.

We validated our approach by performing some fixed walks around the shop, observing that we can trust the reconstructed trajectories as long as the interval between detection does not exceed three minutes.

From this file, assuming that the trajectory built by Lee’s Algorithm is realistic enough, and through simple calculations, we can also extract some numeric features of any trajectory. Some of them are calculated in a straightforward manner from the contents of the data file:

**Entering Time**  Directly extracted from the file and expressed in Coordinated Universal Time (UTC).
**Leaving Time**  Directly extracted from the file. Same format.
**Staying Time**  Calculated as the difference, in seconds, between Entering and Leaving Times.

Both Entering and Leaving Time are converted into a value corresponding to the number of seconds past since the shop opened in the morning. All the variables are normalized using the Z-score method before being used by the clustering algorithm. Some other values are calculated after the generation of full paths:

**Total Path Length**  Defined as the number of red squares (detection points) plus the number of yellow squares (those generated by Lee’s Algorithm). Each tile is a 1 square meter space. See Figure 5.
**Average Speed**  Total Path Length, in meters, divided by Staying Time.
**Detection Points**  Number of red squares in Figure 5. This represents the number of times a customer was detected before leaving the shop.

These magnitudes will be used as basic inputs for the unsupervised clustering algorithm that we will be applying on the data. However, apart from them, we also use second-order values that we calculate studying the trajectories, in terms of the sequence and proportion of different Logistic Sections that the customer is visiting, or the number of times that a person steps on a single tile. These variables are listed below.

**Redundancy**  Percentage of times that the customer steps on the same square. Calculated as the Total Path Length minus the number of times a user steps on a unique 1 × 1 m tile forming the trajectory, divided by Total Path Length.
**Logistic Coverage**  Percentage of Logistic Sections visited by a customer. A Logistic Section is each one of the areas labeled with a different name (i.e., CYCLING, RUNNING, etc.). If the customer visits 6 sections out of 30, this value would be equal to 0.2.
**Logistic Sequence**  We map the list of (x,y) coordinates describing the full path obtained by Lee’s algorithm into a list of Logistic Sections that represents the ordered list of sections that the customer visits, allowing repetition. A full trajectory is then converted into something like
  [1,1,3,6,7,7,7,7,7,1]
  meaning that the user stepped twice on section one, once on sections three and six, five times on section seven, and once again on section one during the walk through the shop. This variable is not used by the clustering algorithm, but it is used to calculate the value of the variable named Logistic Stayings, described as
**Logistic Stayings**  From the *Logistic Sequence* we obtain a list of percentages that is an estimation of the relative amount of time spent by the customer inside each Logistic Section. The list represented above would generate something like
  [0,30,0,10,0,0,10,50,0,0, .......,0]

This sequence of values is motivated by the customer being detected five times out of ten inside section seven, three times out of ten inside section one, and once in sections three and six. The customer is supposed to have spent thirty percent the time in the store in Section 1, ten percent in sections three and six, and fifty percent in section seven. Using this sequence makes it possible to compare trajectories with different lengths (i.e., a different number of (x,y) coordinates), a problem described in [16]. In the same work, authors question the convenience of using this type of variable in a k-means algorithm, but we are using a higher number of logistic sections (30), so the probability of assigning totally different paths to the same cluster is not significant.

**Collapsed Logistic Sequence**  Converting *LogisticSequence* into a list without consecutive duplicates, we obtain, for the same example, the following.
  [1,3,6,7,1]

## 3. Clustering

In a Supervised Learning context a class label would be given for each customer, and based on this label, and on the values of pre-calculated variables, new customers would be classified [24,25]. In our case, the class label of each customer is unknown. Through clustering analysis, these groupings are discovered. Clustering is the process of partitioning a set of data objects into subsets [17]. Each subset is a cluster, and objects in a cluster are similar to one another, yet dissimilar to objects in other clusters. The partitioning is not performed by people, but by the clustering algorithm instead. The correct number of clusters or their definition is not known beforehand. Therefore, different clustering methods could end up generating different clusterings for the same input. Consequently, clustering is useful in that it can lead to the discovery of previously unknown groups within the data. Cluster analysis has been widely used in many applications such as Image Pattern Recognition [26,27], Business Intelligence [28], or Information Retrieval [29,30].

### 3.1. Clustering Algorithm Alternatives

In this section, we discuss five alternatives for the clustering process, describing in detail the option selected and the reasons that inspired this choice.

#### 3.1.1. K-Means Clustering

Based on the works of Stuart Lloyd [31] and J.B. McQueen [32], k-means algorithm is probably the most well-known clustering algorithm. Its main advantage is its linear complexity, O(n) as it is based on distance computations between points to be classified and candidate centroids. On the other hand, its main disadvantage is that the user must decide the number of classes beforehand. This problem can be easily solved using different techniques [17,33] that we will discuss in Section 3.3.

#### 3.1.2. Mean-Shift Clustering

First presented by Fukunaga in 1975 [34], Mean-shift Clustering is a sliding-window-based algorithm that attempts to find dense areas of data points. It builds upon the concept of Kernel Density Estimation or KDE. The goal is to locate the center points of each cluster, updating centroids to be the mean of the points within the sliding window. The main advantage is that the user does not need to set the number of clusters as it can automatically discover it. There are two disadvantages when using mean-shift algorithm:

1. The selection of the window size can be non-trivial in some cases.
2. It is computationally expensive( $O(n^{2})$).

#### 3.1.3. Density-Based Spatial Clustering of Applications with Noise (DBSCAN)

In general, the expression Density-Based Clustering refers to any unsupervised learning method that identifies the clusters present in the data based on the concept that each of such clusters is defined as a region with a high density of examples separated from the others by contiguous regions with a low density of examples. DBSCAN [35] is mainly a variant of a mean-shift algorithm with two additional advantages, and some important disadvantages. The two major features are as follows.

1. It does not require a preconfigured number of clusters.
2. It can sometimes identify outliers as noise, unlike mean-shift which puts them into a separate cluster no matter how different the points are.

The main problems that have been identified in applying this algorithm are twofold, both of them being related to the challenge of estimating the distance threshold, ϵ:

1. It does not perform well when dealing with clusters of varying density. In [36], a variant of this algorithm has recently been presented to solve this problem, but it is computationally expensive.
2. It is not well equipped to deal with high-dimensional data.
3. Depending on implementation details, complexity can vary from O(n×logn) to $O(n^{2})$.

#### 3.1.4. Expectation–Maximization (EM) Clustering Using Gaussian Mixture Models (GMM)

Gaussian mixture models are a form of representing normally distributed subpopulations within an overall population.

To learn the best parameters of the gaussiuans, the EMM algorithm [37,38,39] is used. These methods perform better than classic k-means when clusters overlap, and when examples are normally distributed. On the other hand, its complexity is $O(m\times n^{3})$, being m the number of iterations we want to perform and n the number of parameters.

#### 3.1.5. Agglomerative Hierarchical Clustering (AHC)

Agglomerative Hierarchical Clustering ([40]) works by starting from a set of examples each one belonging to a separate cluster. From this initial situation, a similarity matrix is computed, and the two most similar clusters are fused. The process is repeated until eventually all points belong to a single cluster. This kind of hierarchical clustering algorithms consume a significant amount of memory to store the similarity matrix, and are computationally expensive $O(n^{3})$, being not the most suitable when it comes to handling large volumes of data.

### 3.2. Clustering Process

To conduct a Cluster Analysis, several steps are necessary:

1. Select variables on which to cluster. We used the variables described in Section 2.3.
2. Select a Similarity Measure and scale the variables. We used Euclidean Distance, and normalized all the variables using Z-score. A Z-score is a measure of how many standard deviations below or above the population mean a raw score is, and is frequently used prior to any Data Mining Technique ([41]). This prevents the inherent differences in the absolute values between variables from skewing the analysis.
3. Select a clustering method. In our case the k-means method was chosen, for the following reasons:
  (a) Ease of implementation.
  (b) Scalability. Its linear complexity allows us to use the same methodology with larger datasets.
  (c) Guaranteed convergence.
  (d) It can be easily adapted to new examples. In the case of identifying new variables that may be of use in the clustering process, the adaptation of the algorithm is straightforward.
4. Determine the number of clusters. Using K-means Algorithm permits the automatic determination of the optimal number of clusters using one of two methods. We have used both in the past, choosing the Silhouette Method [42] only when the results obtained from the Elbow Method were not acceptable. In this work we used the Elbow Method, as described in [17,33].
5. Conduct the Cluster Analysis, interpret the results, and apply them.

### 3.3. K-Means Algorithm

K-means is an iterative clustering algorithm that aims to find local maxima in each iteration. This algorithm is a Centroid-Based Technique and belongs to the family of Partitioning Methods. It works in five steps:

1. Specify the desired number of clusters, k. Suppose a data set D contains n objects in Euclidean space. Objects need to be distributed into k clusters, $C_{0}..C_{k-1}$.
2. Randomly assign each data point to a cluster.
3. Compute cluster centroids. A centroid based partitioning technique uses the centroid of a cluster, $C_{i}$, to represent that cluster. The centroid can be defined in various ways such as by the mean or medoid of the objects assigned to the cluster.
4. Re-assign each point to the closest cluster centroid. The difference between an object p belonging to $C_{i}$ and $C_{i}$, the representative of the cluster, is measured by dist(p,Ci), where dist(x,y) is the Euclidean distance between two points, x and y.
5. Re-compute cluster centroids.
6. Repeat steps 4 and 5 until no improvements are possible. If not explicitly mentioned, when there is no switching of objects between two clusters for two successive repeats, the algorithm has finished.

### 3.4. Automatic Determination of the Number of Clusters

Determining the appropriate number of clusters is one of the open problems in non-supervised clustering. Usually, the criteria tend to be subjective. In the context of this work, the size of the clusters should be large enough to be managerially meaningful. If a cluster contains few objects (paths, or trajectories), it should be treated as containing mainly outliers, and could be ignored. One method to validate the number of clusters is the Elbow Method [17,33]. The idea is to run k-means clustering on the dataset for a range of values of k, calculating the Sum of Squared Errors (SSE) for each value of k.

Plotting a line chart of the SSE for each value of k, the chart usually looks like an arm. If this is the case, the elbow on the arm is the best possible value of k for the data set. We assume that small SSE is better, but it tends to decrease toward 0 as the value of k rises (the SSE is 0 when k is equal to the number of data points in the dataset, because then each data point is its own cluster, and there is no error between it and the center of its cluster). As a general rule, the goal is to obtain a small SSE with a small, and meaningful number of clusters.

**Figure 6:**
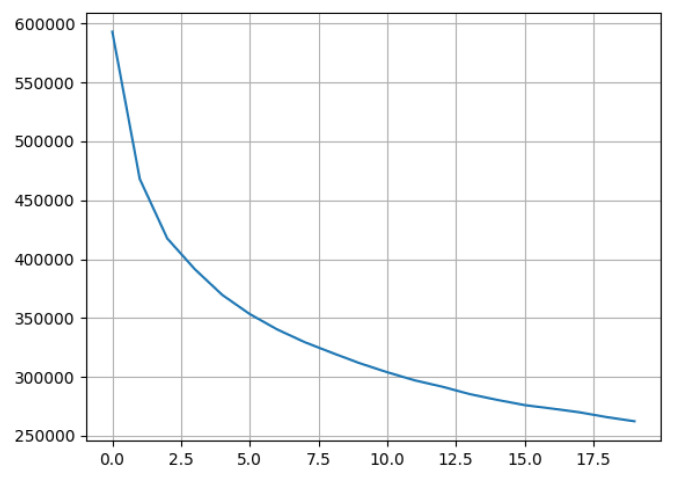
Elbow inertial method.

For our test data set, after applying the Elbow Inertial Method, a theoretical approach would suggest to identify a number of clusters between 5 and 10, as can be seen in Figure 6, showing results up to 20 clusters. In order to increase the readability of the results, we have chosen the minimum number of clusters indicated by the Elbow Method. Nevertheless, the number of clusters can be configured in the same way as the period under study, as what we are defining is an agile methodology for assessing the dynamics of the establishments. In Section 4, we summarize the results when running the whole system on data corresponding to one month of commercial activity.

## 4. Results

In this section, we present and discuss the results obtained when applying k-means Algorithm on a data set formed by trajectories corresponding to one month of commercial activity. During this period we registered a total number of 1368 visits, but we cannot state the exact number of different people this number corresponds to, as their MAC addresses are hidden for us. For such a period, we received over 40,000 readings, with individual visits containing a number of positioning marks between 10 and 64. See Table 1 for details. In other studies, such as that in [16], the size of the data set is greater, but our goal was to define a methodology and to develop a tool that can operate on any desired data set, no matter its size. Solely by selecting the appropriate records from the database, we can study the dynamics of the store during the current week, just for all the Mondays of September, or for a random choice of days. To illustrate the utility of this approach, we split the original set of trajectories into two different sets: one for the regular week days (930 trajectories), and the other for the weekends (438 trajectories), and compare the results with those obtained from the whole set, and between them. As previously mentioned, we conducted a non-supervised clustering process setting k to a value of 5.

We have divided the analysis of the results into two parts:

- Visual analysis of the clusters that the system has identified.
- Numerical analysis of the characteristics of each cluster.

All the conclusions in this study are extracted from the observation of heatmaps.

**Heat Map:**  A heat map is a two-dimensional representation of information with the help of colors. In our case, each point representing a 1 × 1 square meter in the shop is plotted proportionally as red as how many times a user stepped on it, according to the paths generated by Lee’s Algorithm. We will use heat maps as a tool to visually identify the areas of the shop that attract more interest over a fixed period.

### 4.1. Visual Interpretation of Heat Maps

In this section we visually analyze the data concerning the trajectories, with and without clustering.

#### 4.1.1. Non Clustered Heat Maps

**Figure 7:**
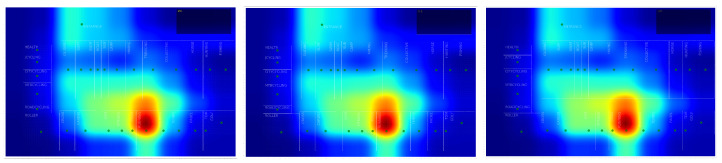
General heat map (**left**). Week (**center**). Weekends (**right**).

We can see some valuable information in Figure 7 (left), where a general (non-clustered) heat map is represented. It is clearly biased by the existence of only one main entrance and exit, so there is a “heated up” area close to it. Checkout lanes are also in this neighborhood. All the customers must pass through this zone when entering and leaving the shop, so knowing that this is one of the most visited areas can tell us that our measurements are correctly taken, but says nothing about the real deep dynamics of the shop. The observation of the general non-clustered heat map contradicts the racetrack theory as described in [16] (myth about people spending most of their time moving along the outer ring of the shop), showing a more or less straight general trajectory to section named RUNNING and its neighbors. However, because this is partially true, as the RUNNING section is traditionally the most crowded section at any time, this heat map is clearly influenced by the store’s layout.

The same type of behavior is observed when we plot non-clustered heat map for Week days (see Figure 7 (center)), and for the weekends (Figure 7, right). Although presenting some slight differences at some areas, these three heat maps are quite similar, so we can state that there was no remarkable difference between week and weekend days, in terms of customer’s physical behavior. Clustering will reveal different areas of interest that remain hidden in the general heat map, as we will see in following sections.

#### 4.1.2. Detailed Heat Maps after Clustering

**Figure 8:**
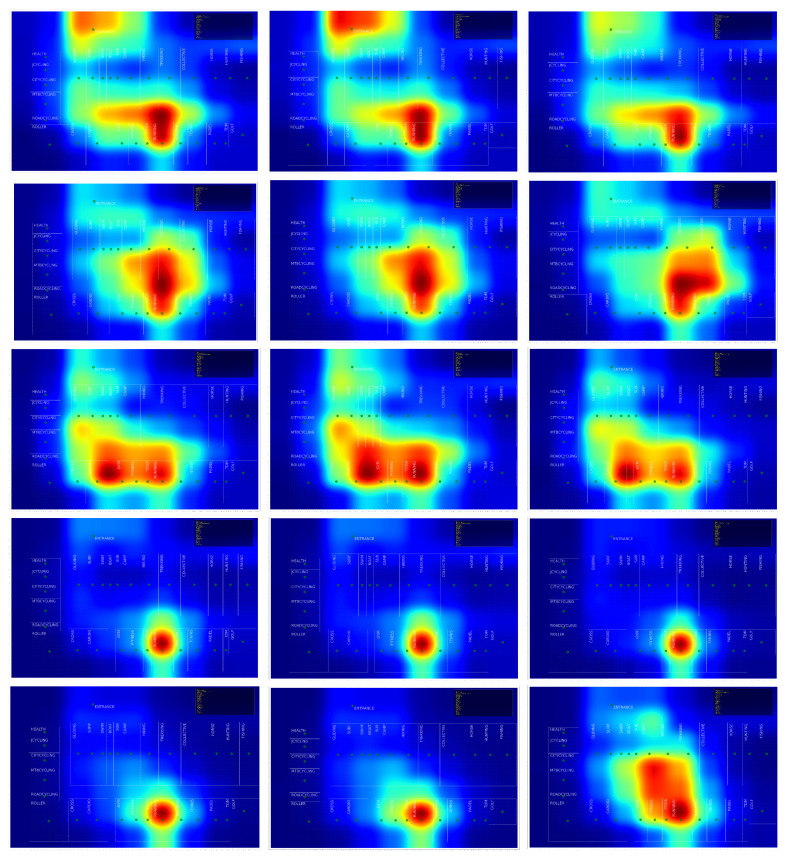
Detailed results for classes 0 to 4. The whole set of trajectories is on the left. Weekdays are in the center, and weekends on the right.

Due to the random nature of the clustering process, we cannot guarantee that what is called Class 0 when we perform the experiment on the total set of trajectories will also be labeled as Class 0 on the normal set of weekdays, or on the set of weekends. Therefore, we have rearranged the images in Figure 8, putting the most similar clusters in the same row for each of the three experiments. Therefore, when referring to the figure, the first row corresponds to what we will call Class or Cluster 0, independently of the number that the clustering process has assigned to it, the second row will be class 1, and so on up to the fifth row, which corresponds to class 4.

In view of the results, it could be argued that the fifth cluster is unnecessary for the trajectories corresponding to weekdays (second column of the Figure 8). However, the analysis of the trajectories belonging to the weekends reveals the existence of a fifth cluster whose clients have a significantly different behavior.

Class 0 is quite similar in all three experiments, although it could be deduced that the transit through the cash-desk, input and output zones is more fluid during the weekends, as that zone seems to be less “burned out” on the maps. See first row in Figure 8.

Class 1, corresponding to the second row, is very similar in the overall set of trajectories and in the one corresponding to weekdays, covering a slightly larger area at weekends.

Class 2 has no significant differences in the three experiments conducted.

The first conclusion we can draw is that we have segmented a type of customer which is present only during weekend. See Figure 8, bottom, right.

### 4.2. Numerical Study of the Clustered Data

We have analyzed the results of the clustering process according to the three variables that best define client behavior:

- Distribution of the customers depending on the cluster (Table 2).
- Average visit time per cluster (Table 3).
- Logistic coverage, i.e., percentage of sections visited by customers inside each class (Table 4).

In the three experiments carried out, the distribution of the clientele by clusters proved to be practically equivalent, except for the greater presence of Class 0 clients in the weekend trajectories, where it reached a third of the visits as opposed to 20 percent on weekdays.

Classes 0, 1, and 2 appear in all three experiments with similar behavior, time spent in the same range, and similar representativeness (weight of each cluster), except for a greater presence of class 0 at weekends (33% compared to 24% and 21%).

As far as the difference between Classes 3 and 4 is concerned: both focus on the same area of the shop, but Class 3 is a significantly shorter type of visit on average (34 min compared to 40 for the whole shop, 33 min compared to 44 on weekdays). At weekends, a new type of tour appears that cannot be assimilated to any of the other sets: class 5. In class 4, and for weekends, the time spent in the shop is slightly longer (37 min as opposed to 33 and 34).

## 5. Discussion

Different types of buyers will move around the shop in substantially different ways, showing interest in different groups of products, so Path Reconstruction can be useful when performing an Automatic Segmentation of Customers. Using only the position signal that we can obtain from mobile phones, tablets, and any other device that connects to our facilities’ network, we have developed a tool that is able to process existing data about customers’ paths, for a chosen period of time, automatically clustering them in a way that is managerially meaningful. Customer behavior changes frequently, depending on factors such us the weather, economy, and holiday periods, so performing cluster-based segmentation just occasionally is not sufficient. We can perform this analysis on a daily basis and also monitor the real time state of the shop at any moment, taking advantage of all the latest customer behavioral data.

We assume that customers keep their WiFi interfaces up on their mobile phones, and that this will give us a detection rate around 80%, according to recent studies. This is a limitation of our method as we will miss spatio-temporal information about the rest of the users, but still with a higher detection rate than those studies using Bluetooth technologies [15].

Applying this data process we obtain very relevant information regarding the following.

- Determination of Store Operational Requirements by scheduling and assigning employees. By analyzing the pattern of visits at specific periods, it is possible to reallocate personnel to respond to possible peaks in the activity.
- All the knowledge that can be extracted about customer behavior patterns is of great value in personnel selection and training processes.
- Identification of current and future customer requirements. The use of our tool on an ongoing basis allows early detection of changes in customer behavior.

## 6. Conclusions and Future Work

The conclusions presented in this paper are valid for the set of data that we were studying at the moment of writing it. Actually, our system is a tool conceived and designed to be used on a daily basis, as consumer behavior can change depending on socioeconomic factors as the day of the month, the proximity of holidays, or even sport events, as we are dealing with data coming from a huge sport center. For that reason, some of the variables used in the study can seem useless, but it is also true that this happens only for the data set reported here. A forthcoming study is based on Redundancy, in order to search for changes in the store’s layout that can minimize the length of the paths, and visiting time, as this factors are usually related to customer’s satisfaction and return willingness. The fundamental conclusion of this study is the possibility of extracting relevant knowledge about the habits of the customers of a large shopping center based only on the information that can be deduced from their movements within the facilities. The interpretation of this data, and its possible usefulness from a logistic point of view, is not part of the study itself, but belongs to the business field. It has also been proven that automatic clustering is useful in areas such as this, where there is a large volume of information that was not accessible until the arrival of intelligent mobile devices.

In the near future we are planning to compare trajectory clustering with actual purchase data. This information is not available at the moment, but is of great interest in order to analyze relationships between what people do physically, and what they really buy. This study is also of interest when planning any kind of physical reallocation of Logistic Sections within the premises.

We are also studying the possibility of using the information that we collect to generate Sankey diagrams, and the results of the clustering process to train our system to perform trajectory predictions, in a way different from those proposed in [43,44,45,46]. In this sense, we think we can use our logistic sequences to predict the whole trajectory of a customer as soon as our tracking system has registered only a few points. Having segmented customers as a previous step can help to train different forecasting routines for each cluster.

## 7. Materials and Methods

Due to privacy consideration regarding subjects in our dataset, including European Union regulations and Spanish Data Protection Agency rules, we cannot make our data publicly available. We understand and appreciate the need for transparency in research and are ready to make the data available to researchers who meet the criteria for access to confidential data, sign a confidentiality agreement, and agree to work under our supervision. Please direct your queries to Dr Santiago García Carbajal, the Principal Investigator of the study, at sgarcia@uniovi.es.

## Figures and Tables

**Table 1:** Total number of positional marks and trajectories over the studied period.

Readings	Detection Points per Visit		Trajectories
40,408	min:11	max:64	1368

**Table 2:** Detailed results. Cluster distribution. Whole set, week days, and weekends.

Class	Whole Set	Week Days	Weekends
0	24%	21%	33%
1	19%	19%	15%
2	25%	27%	24%
3	16%	16%	15%
4	13%	15%	12%

**Table 3:** Detailed results. Average visit time, in seconds. Whole set, week days and weekends.

Class	Whole Set	Week Days	Weekends
0	2445	2237	2346
1	4089	4095	4004
2	3475	3564	3429
3	2055	1990	2242
4	2449	2674	3065

**Table 4:** Detailed results. Logistic coverage. Whole set, week days and weekends.

Class	Whole Set	Week Days	Weekends
0	24%	23%	24%
1	28%	25%	25%
2	28%	29%	25%
3	22%	22%	21%
4	22%	22%	22%

## Data Availability

Not applicable.

## Abbreviations

The following abbreviations are used in this manuscript:

AHC	Agglomerative Hierarchical Clustering
AP	Access Points
DBSCAN	Density-Based Spatial Clustering of Applications with Noise
EM	Expectation–Maximization
FSPL	Free Space Path Loss
GMM	Gaussian Mixture Models
KDE	Kernel Density Estimation
MAC	Media Access Control
RSSI	Received Signal Strength Indication
SSE	Sum of Squared Errors
VLSI	Very Large Scale Integrated circuit
WPS	WiFi Positioning System
